# Preoperative Gabapentin in Pediatric Anterior Cruciate Ligament Reconstruction With Peripheral Nerve Blockade: A Retrospective Cohort Study

**DOI:** 10.7759/cureus.113552

**Published:** 2026-07-28

**Authors:** Lloyd M Halpern, Charlotte Corell, K. Perrins, De-An Zhang, Kirk Ryan, Jacob R Carl

**Affiliations:** 1 Pediatric Anesthesia, Shriners Children's Spokane, Spokane, USA; 2 Anesthesiology, University of Utah Health Care, Salt Lake City, USA; 3 Anesthesiology, University of Florida Health Science Center Jacksonville, Gainesville, USA; 4 Pediatric Anesthesia, Shriners Children's Southern California, Pasadena, USA; 5 Anesthesia, Shriners Children's Spokane, Spokane, USA; 6 Orthopedics, Shriners Children's Spokane, Spokane, USA

**Keywords:** anterior cruciate ligament reconstruction (aclr), oral gabapentin, pediatric, peripheral nerve block analgesia, post-operative opioid consumption, postoperative pain

## Abstract

Background

Preoperative gabapentin has been shown to reduce postoperative pain and opioid consumption following anterior cruciate ligament reconstruction (ACLR) in adult patients treated with local infiltration analgesia. Its effectiveness in pediatric patients receiving peripheral nerve blockade for ACLR remains unclear.

Methods

We conducted a retrospective, single-center cohort study of pediatric patients (<18 years) undergoing ACLR from 2019 to 2024. All patients received anterior sciatic and adductor canal peripheral nerve blocks. Patients were stratified by receipt of preoperative gabapentin. The primary outcome was total postoperative opioid usage prior to hospital discharge (morphine milligram equivalents, MME). Secondary outcomes included pain scores, sedation, block efficacy, and length of stay. Multivariable linear regression was performed adjusting for patient demographics, intraoperative opioid usage, anesthesiologist, and tourniquet time.

Results

A total of 79 patients were enrolled in the study, with 55 in the Gabapentin group and 24 in the No Gabapentin group. Postoperative opioid consumption was low and not significantly different between groups (Gabapentin 4.1 ± 9.2 vs No Gabapentin 5.2 ± 5.7 MME, p=0.08). In adjusted analysis, preoperative gabapentin was not associated with postoperative opioid use (β = -0.56, p=0.25). Pain scores, sedation, block efficacy, and length of stay were similar between groups. Intraoperative opioid use was significantly higher in the Gabapentin group (p<0.001). Nearly one-third of patients required no postoperative opioids prior to discharge.

Conclusions

Preoperative gabapentin was not associated with a detectable reduction in postoperative opioid use or improvement in pain control in pediatric ACLR patients receiving adductor canal and anterior sciatic peripheral nerve blocks. When highly effective regional anesthesia is employed, gabapentin may provide limited incremental benefit.

## Introduction

The incidence of anterior cruciate ligament reconstruction (ACLR) has increased 148% in the past decade in 14-18-year-olds, paralleling increased sports participation, early sport specialization, and developmental biomechanical risk factors [1].

ACLR is associated with substantial postoperative pain due to soft tissue dissection and drilling of distal femur and proximal tibia tunnels. Optimizing perioperative analgesia while minimizing opioid exposure remains a clinical priority in pediatric orthopedic care. Two prospective, randomized clinical trials found an opioid-sparing effect of a single dose of gabapentin (600 mg or 1200 mg) in adult patients with local infiltration analgesia [2,3]. However, the American Academy of Orthopedic Surgeons does not recommend the routine use of preoperative gabapentin for ACLR in adults secondary to an increased risk of sedation, dizziness, and respiratory depression when combined with opioids [4]. In contrast, contemporary pediatric ACLR practice frequently incorporates peripheral nerve blockade, most commonly adductor canal blocks, with adjunct sciatic blockade increasingly utilized [5].

Combined peripheral nerve blockade provides highly effective analgesia for pediatric ACLR; hence, the incremental benefit of gabapentin may be limited. To our knowledge, there are no previous studies evaluating preoperative gabapentin for ACLR in pediatric patients. Therefore, we conducted a retrospective cohort study to determine whether preoperative gabapentin reduces postoperative opioid use and improves pain control in pediatric patients undergoing ACLR with continuous adductor canal and single injection anterior sciatic nerve blockade. The primary endpoint is opioid usage in the postoperative period. Secondary endpoints include pain scores, sedation, length of stay, and block failure rate. We hypothesized that gabapentin would not provide a clinically meaningful reduction in opioid requirements.

## Materials and methods

We conducted a retrospective cohort study of pediatric patients who underwent primary ACLR with peripheral nerve blockade between 2019 and 2024 at a single pediatric orthopedic hospital, Shriners Children's Spokane, Spokane, Washington, United States. This study was reviewed and approved by the WCG institutional review board, accredited by the Association for the Accreditation of Human Research Protection Programs (approval number: 1394165). In accordance with United States federal regulations (45 CFR 46.116), the study was granted a waiver of informed consent given that the study posed minimal risk and involved a retrospective review of medical records. Data access and use were conducted under institutional data use agreements with the contributing health system.

Study population

Consecutive pediatric patients aged 18 years or less who underwent primary ACLR with peripheral nerve blockade between 2019 and 2024 at XX were included in the study. Patients who underwent additional procedures on the same knee (e.g., meniscal repair) were included. Patients were excluded if they had concomitant procedures beyond the operative knee, revision ACLR, preexisting neurosensory deficit, or an inability to provide verbal pain scores. A formal sample size calculation was not performed. All eligible patients during the study period were included to minimize selection bias.

Surgery and analgesia process

As part of a multimodal pain management strategy, pediatric anesthesiologists (six) had the option of prescribing a single dose of gabapentin (5 mg/kg) administered within two hours of the procedure, creating two groups: Gabapentin and No Gabapentin.

All patients underwent an arthroscopically assisted ACLR using quadriceps tendon autograft by one of two pediatric orthopedic surgeons. All patients had combined anterior sciatic and continuous adductor canal blocks placed after induction of general anesthesia, and multimodal analgesia with acetaminophen (15 mg/kg) and ketorolac (0.5 mg/kg). Intraoperative opioid dosing was at the discretion of the anesthesiologist (morphine and fentanyl). Postoperatively, all patients had intravenous (morphine) and oral (oxycodone) opioids available as needed for a pain score of four or more. Hydromorphone and hydrocodone were available when these were not tolerated. The surgeons elected to have some patients observed overnight for observation when indicated, including for pain control or late hours at discharge.

**Figure 1:**
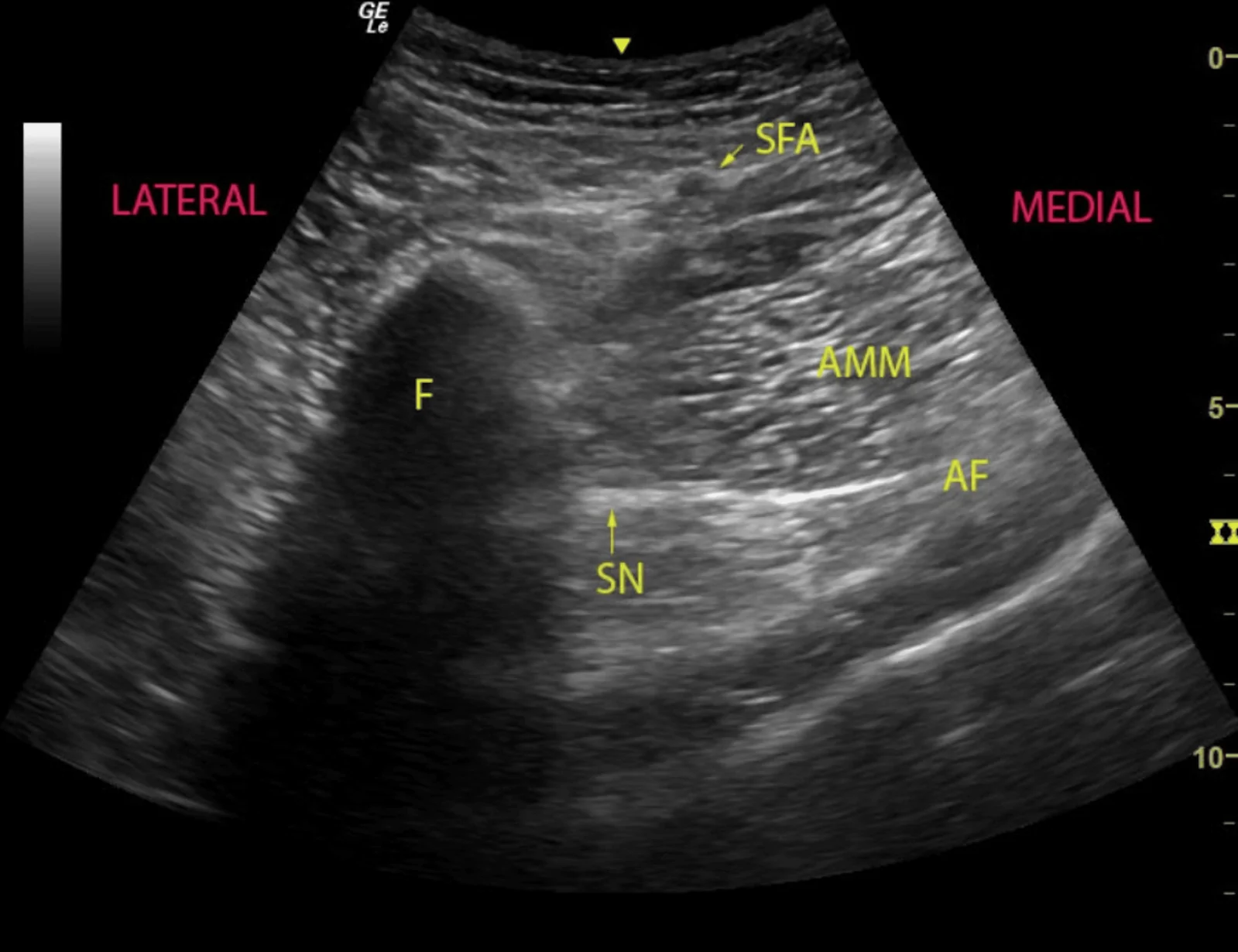
Ultrasound image before needle placement. The curved array transducer is placed in the proximal third of the thigh medial to the femur at the level of the proximal adductor canal block. The sciatic nerve (SN) is well visualized 2-3 cm medial to the femur (F) and inferior to the adductor fascia (AF).  Note the superficial femoral artery (SFA) which must be visualized prior to needle advancement. AMM, adductor magnus muscle Source: Halpern et al., 2022 [6]; reproduced with permission from Taylor & Francis Informa UK Ltd.

All patients underwent anterior sciatic and continuous adductor canal blocks under general anesthesia in the supine position using ultrasound guidance (LOGIQ™ e; GE Healthcare Technologies, Inc., Chicago, Illinois, United States), as described in our previously published study [6]. The anterior sciatic block was performed as a single injection in the proximal thigh with a curved probe (C1-5-D Curved Array; GE Healthcare Technologies, Inc.). The sciatic nerve was identified 2-4 cm medial and posterior to the femur beneath the adductor magnus and fascia (Figure 1).

**Figure 2:**
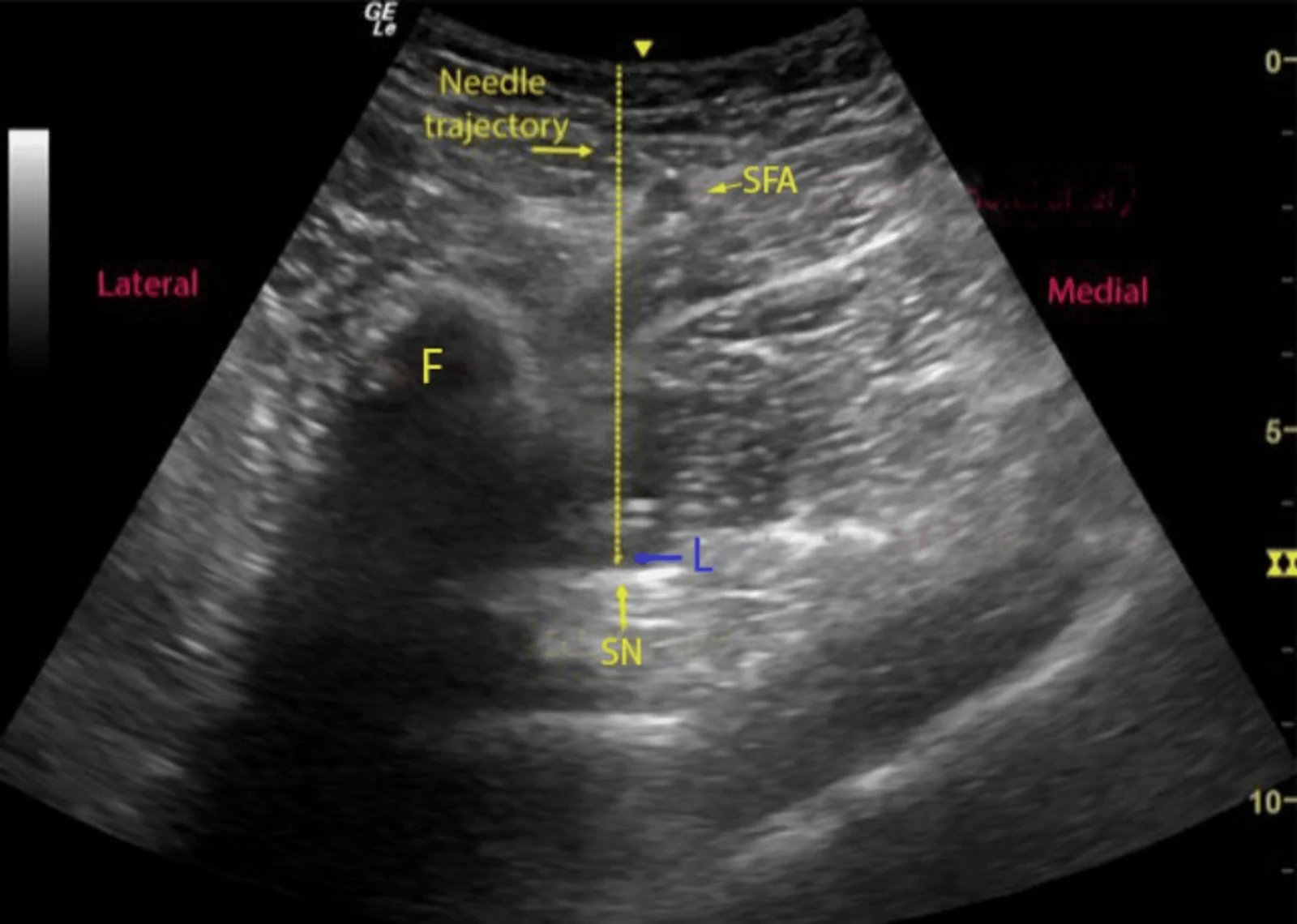
The ultrasound image immediately after local anesthetic injection. Note the local anesthetic (L) inferior to the adductor fascia. The needled trajectory is demonstrated (dashed line) The sciatic nerve (SN) is visualized within the local anesthetic and has moved inferiorly from the adductor fascia. Source: Halpern et al., 2022 [6]; reproduced with permission from Taylor & Francis Informa UK Ltd.

An out-of-plane needle approach was used, avoiding the superficial femoral artery. Nerve stimulation (2 mA, 0.10 ms, 2 Hz) confirmed placement via plantar flexion or hamstring contraction (Figure 2).

Continuous adductor canal blocks were placed with a high-frequency linear probe (L4-12T-RS Wideband Linear Array; GE Healthcare Technologies, Inc.). The femoral artery was visualized medial to the sartorius at the femoral triangle apex. After initial injection, a catheter was advanced perpendicular to the arteru1-3 cm beyond the needle and secured. Infusions of plain 0.2% ropivacaine were started in the post-anesthesia care unit (PACU) at 0.1 mL/kg/hr (maximum, 8 mL/hour) and maintained for 48-72 hours using an Ambu® ACTion™ Fuser Pain Pump (Ambu A/S, Copenhagen, Denmark). Patients removed the catheter at home. Total dose was 2.0 mg/kg of 0.5% ropivacaine (max 30 mL), split between sites, with dexamethasone (2 mg) and dexmedetomidine (50 mcg).

Data collection

We collected demographic data, operative variables, tourniquet time, anesthesiologist, surgeon, and opioid usage intraoperatively, in the PACU (Phase 1) and until hospital discharge from the inpatient floor (Phase 2). All parenteral and oral opioids were converted into morphine milligram equivalent doses (MME) using standard opioid conversion tables [7]. The primary outcome was unadjusted total postoperative opioid use from PACU admission through hospital discharge. Secondary outcomes included numeric pain scores, Aldrete sedation scores in Phase 1, incidence of nausea and vomiting, pruritus, block failure rate (defined as a pain score greater than or equal to five in Phase 1 or 2 recovery), and the block rate of complete analgesia (all pain scores 0 and no opioids postoperatively prior to discharge). All clinical outcome data (pain scores, sedation scores, opioid usage, nausea, and pruritus) were extracted from the electronic medical records (EMRs).

Data analysis

To assess the study’s ability to detect meaningful differences in MME between groups, a post hoc sensitivity analysis was conducted using the mean MME for each group and the pooled standard deviation (SD) of total MME. With 55 patients in the Gabapentin group and 24 patients in the No Gabapentin group, the study had approximately 0.66 power to detect a difference of 7.0 MME, corresponding to a 40% difference relative to the Gabapentin group. For a future prospective study, assuming a 20% difference as the minimum clinically important difference, an estimated total sample size of 360 patients (180 patients per group) would be required to achieve 80% power at a two-sided significance level of 0.05.

Descriptive statistics were used to summarize patient demographics and clinical characteristics. Categorical variables were reported as frequencies and percentages, and continuous variables were summarized as mean ± SD for variables with approximately normal distributions (age, height, weight, BMI, and MME). Between-group comparisons (Gabapentin vs No Gabapentin) were performed using chi-square tests or Fisher’s exact tests for cell counts less than five. Continuous variables were analyzed using two-sample t tests or Wilcoxon rank-sum tests, as appropriate based on whether values were normally distributed. All analyses were performed using SAS 9.4 (Released 2023; SAS Institute Inc., Cary, North Carolina, United States). Statistical tests were two-sided, and p-values less than 0.05 were considered statistically significant. Multivariable linear regression was performed with log-transformed postoperative MME as the dependent variable. Covariates included gabapentin use, intraoperative MME, age, sex, BMI, tourniquet time, and anesthesiologist (modeled as a categorical variable).

## Results

A total of 79 patients met inclusion criteria. Of these, 55 were in the Gabapentin group, and 24 were in the No Gabapentin group. The mean dose of preoperative gabapentin was 298 mg (± 87.2) (mean 4.4 mg/kg). Demographic and surgical characteristics were similar between groups apart from anesthesiologist distribution, which differed significantly (Table 1).

**Table 1:** Demographic data in the two treatment groups. * p<0.05; ** Surgeons were not included in the covariates because the numbers were so one-sided. C, Caucasian; AA, African American; O, Other

Variable	Gabapentin (n=55)	No Gabapentin (n=24)	p Value
Age (years), mean ± SD	14.9 ± 2.2	14.8 ± 2.8	0.96
Gender			0.48
Female	25 (45.4%)	13 (54.2%)	
Male	30 (54.6%)	11 (45.8%)	
Weight (Kg), mean ± SD	67.7 ± 17.7	63.3 ± 17.4	0.31
BMI (kg/m2), mean ± SD	23.9 ± 4.6)	23.2 ± 4.4	0.49
Race (C/AA/O)	43/2/10	20/2/2	0.40
Surgery Duration (minutes), mean ± SD	201.0 ± 47.8	199.1 ± 45.8	0.85
Tourniquet time (minutes), mean ± SD	86.4 ± 2.4	91.8 ± 1.8	0.11
Surgeon (JC/BT)**, n (%)	51 (92.7%) / 4 (7.3%)	20 (83.3%) / 4 (16.7%)	0.24
Anesthesiologist (H-T-M-V-B-R), n (%)	3 (5.4%) - 12 (21.8%) - 15 (27.3%) -13 (23.6) - 7 (12.7%) - 5 (9.0%)	20 (83.3%) - 1 (4.2%) -1 (4.2%) - 1 (4.2%) -1 (4.2%) - 0	<0.0001*

**Figure 3:**
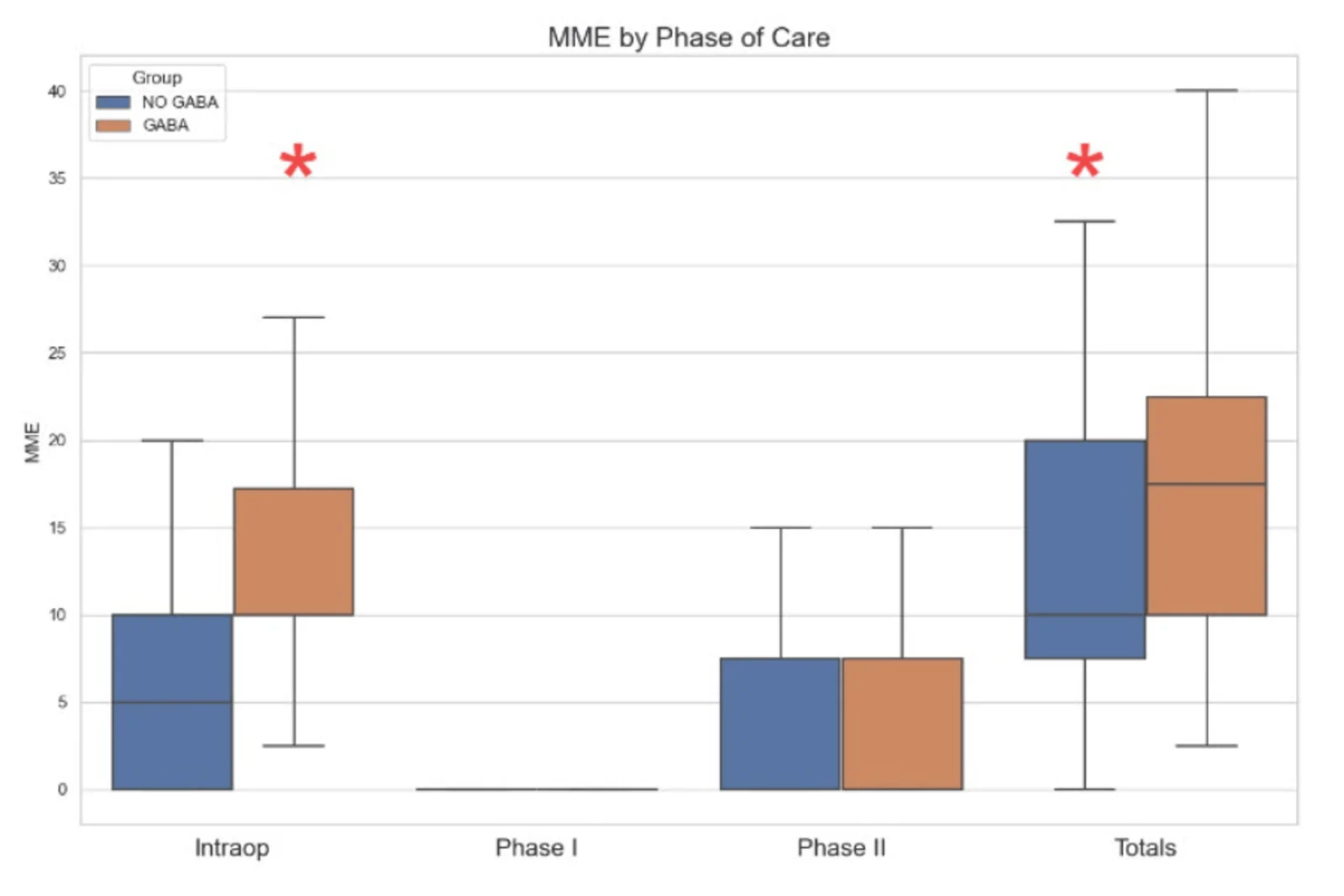
Opioid (MME) usage by phase of care in the Gabapentin and No Gabapentin groups. MME: morphine milligram equivalents; Intraop: Intraoperative; Phase 1:  Post anesthesia care unit; Phase 2: Step-down unit * P<0.05

For the primary outcome, postoperative opioid usage was low in both groups and not significantly different between groups in unadjusted analysis. (MME: Gabapentin 4.1 ± 9.2 vs No Gabapentin 5.2 ± 5.7; p=0.08) In contrast, the Gabapentin group received significantly more intraoperative opioids (13.8 ± 6.7 vs 5.3 ± 6.6 MME, p<0.0001), resulting in greater total perioperative opioid administration (Table 2 and Figure 3).

**Table 2:** Opioid usage (MME) by phase of care in the Gabapentin and No Gabapentin groups. MME: morphine milligram equivalents * P<0.05

Phase of care	Intraoperative	Phase 1	Phase 2	Total Opioids
Gabapentin (SD)	13.8 (6.7)	0.1 (0.5)	4.0 (9.1)	17.4 (13.0)
No Gabapentin (SD)	5.3 (6.6)	0.1 (0.5)	5.1 (5.7)	10.5 (8.2)
p Value	<0.0001*	0.67	0.10	0.002*

Pain scores were low in both groups, with mean and maximum scores in the mild range (<4) during both phases of recovery. No significant differences were observed in pain scores, sedation, or length of stay (Table 3). Ten patients (30.3%) in the Gabapentin group and 19 patients (28.3%) in the No Gabapentin group were admitted for overnight observation by the attending orthopedic surgeon (p=0.84). Nine patients reported nausea or vomiting in the Gabapentin group (16.4%) and seven in the No Gabapentin group (21.2%) (p=0.11). One patient received treatment for pruritus in the Gabapentin group. Dizziness was not reported.

**Table 3:** Mean pain scores, Aldrete sedation scores, and length of stay by phase of care in the two groups. LOS: length of stay

Treatment group	Gabapentin	No Gabapentin	p value
Max Pain Phase 1 (SD)	2.8 (3.1)	1.8 (2.7)	0.11
Mean Pain Phase 1(SD)	2.4 (2.8)	1.6 (2.5)	0.12
Max Pain Phase 2 (SD)	3.1 (3.0)	4.0 (3.1)	0.23
Mean Pain Phase 2 (SD)	1.9 (2.1)	2.0 (2.0)	0.49
Mean Sedation Phase 1 (SD)	1.1 (0.4)	1.3 (0.6)	0.34
Mean LOS Phase 1 (min) (SD)	42.5 (17.9)	36.5 (16.5)	0.08
Mean LOS Phase 2 (min) (SD)	528.5 (477.5)	545.8 (480.9)	0.86

The block failure rates (pain score > 5) were similar between groups (20% vs 25%, p = 0.77), as were rates of complete analgesia (29.2% vs 27.3%, p = 0.76). In multivariable linear regression analysis, preoperative gabapentin was not independently associated with postoperative opioid use (β = -0.56, p = 0.25) (Table 4). BMI was associated with higher postoperative opioid use but did not reach statistical significance (p=0.06).

**Table 4:** Multivariable linear regression analysis of postoperative opioid use. MME: morphine milligram equivalents

Variable	β coefficient	Standard Error	P value
Gabapentin (Y/N)	-0.56	0.48	0.25
Intraoperative MME	-0.01	0.03	0.70
Age (Years)	0.01	0.06	0.93
Sex (Male/Female)	0.07	0.30	0.81
BMI	0.07	0.04	0.06
Tourniquet Time (Hour)	-0.01	0.18	0.96
Anesthesiologist	-	-	0.24

## Discussion

The primary finding of this study is that preoperative gabapentin was not associated with a detectable reduction in postoperative opioid consumption or improvements in pain control in pediatric patients undergoing ACLR with combined anterior sciatic and continuous adductor canal peripheral nerve blocks. These findings remained consistent after adjustment for intraoperative opioid use, patient characteristics, anesthesiologist, and tourniquet time. Additionally, gabapentin did not influence block efficacy, recovery time, or length of stay.

Our findings contrast with prior studies demonstrating reductions in opioid use and pain scores with preoperative gabapentin following ACLR [8]. However, those studies were conducted in adult patients receiving local infiltration analgesia rather than peripheral nerve blockade. A more recent study found preoperative gabapentin with a concomitant adductor canal block did not reduce pain scores or opioid usage in adults 24-72 hours after ACLR [9]. However, they did not compare in-hospital opioid usage or pain scores when the peripheral nerve block was most active. In the present study, pain scores were low, and a substantial proportion of patients required minimal or no opioids prior to hospital discharge, suggesting that this regional anesthesia strategy provides highly effective analgesia. A ceiling effect likely exists, limiting the ability of medications such as gabapentin to provide additional benefit. Notably, nearly one-third of patients achieved complete analgesia with no postoperative pain or opioid requirements prior to discharge. 

The evidence for preoperative gabapentin in pediatric patients is incomplete. Current evidence does not support the routine use of gabapentin in perioperative pain protocols for children [10]. While some studies show modest benefits, primarily following posterior spinal fusion and thoracic surgery [11,12], the overall body of evidence is limited by heterogeneous methodologies and conflicting results. This study supports these recommendations, with preoperative gabapentin providing no detectable improvement in analgesia for ACLR.

The increased use of opioids intraoperatively in the Gabapentin group was a surprise finding and suggests a provider-level prescribing bias, as anesthesiologists who elected to administer gabapentin may also have been more inclined to administer intraoperative opioids. Although we adjusted for anesthesiologist and intraoperative opioid use in multivariable modeling, residual confounding due to provider behavior remains possible. Future prospective studies should incorporate standardized protocols or account for provider-level effects when evaluating perioperative analgesic strategies.

This study has several important limitations. Its retrospective design introduces potential selection bias, as gabapentin administration was not randomized and was strongly associated with individual anesthesiologist practice patterns. The observed opioid requirements were substantially lower than reported in previous adult studies [2,3], suggesting the study may be underpowered to detect small differences in opioid usage between groups. Postoperative opioid use was assessed only through hospital discharge, and the effect of gabapentin on outpatient pain and opioid consumption, when pain may become clinically significant when the blocks wear off, was not evaluated. The gabapentin dose used (approximately 5 mg/kg) was lower than doses commonly studied in adults, and it is possible that higher dosing strategies may yield different results. The use of a dual-block technique may limit generalizability to centers not employing similar regional anesthesia techniques. Finally, all procedures were performed using quadriceps tendon autograft, which may limit applicability to other graft types. Despite these limitations, this study has important clinical implications. The combination of anterior sciatic and continuous adductor canal blockade provided highly effective regional anesthesia, resulting in low opioid requirements across both groups. In this setting, the routine use of gabapentin may offer limited incremental benefit and may represent unnecessary medication exposure with its known risks of sedation and dizziness when combined with opioids.

## Conclusions

Preoperative gabapentin was not associated with improved pain control or reduced opioid usage in pediatric ACLR patients receiving combined continuous adductor canal and single-injection sciatic nerve blockade. The combination of blocks produced exceptionally low postoperative opioid requirements, potentially limiting the ability of gabapentin to demonstrate additional benefit. These findings support the use of regional anesthesia as the cornerstone of multimodal analgesia in this population. Prospective, randomized, controlled studies are needed to confirm these findings and better define the optimal multimodal analgesic protocols for pediatric ACLR.
